# Fluid dynamic design for mitigating undesired cell effects and its application to testis cell response testing to endocrine disruptors

**DOI:** 10.1186/s13036-023-00369-1

**Published:** 2023-08-07

**Authors:** Seungjin Lee, Jinseop Ahn, Seok-Man Kim, Daehan Kim, Jiun Yeom, Jeongmok Kim, Joong Yull Park, Buom-Yong Ryu

**Affiliations:** 1 School of Mechanical Engineering, College of Engineering, Chung-Ang University, Seoul, 06974 Republic of Korea; 2 Present address: Columbia Center for Translational Immunology, Department of Medicine, Columbia University Irving Medical Center, New York, NY 10032 USA; 3 Department of Animal Science and Technology, BET Research Institute, Chung-Ang University, Anseong, 17546 Republic of Korea; 4 Department of Intelligent Energy and Industry, Graduate School, Chung-Ang University, Seoul, 06974 Republic of Korea

**Keywords:** Microfluidics, Human error, Computational fluid dynamics (CFD), Diffusion gradient, Bisphenol-A (BPA), GC-1 cell

## Abstract

Microfluidic devices have emerged as powerful tools for cell-based experiments, offering a controlled microenvironment that mimic the conditions within the body. Numerous cell experiment studies have successfully utilized microfluidic channels to achieve various new scientific discoveries. However, it has been often overlooked that undesired and unnoticed propagation of cellular molecules in such bio-microfluidic channel systems can have a negative impact on the experimental results. Thus, more careful designing is required to minimize such unwanted issues through deeper understanding and careful control of chemically and physically predominant factors at the microscopic scale. In this paper, we introduce a new approach to improve microfluidic channel design, specifically targeting the mitigation of the aforementioned challenges. To minimize the occurrence of undesired cell positioning upstream from the main test section where a concentration gradient field locates, an additional narrow port structure was devised between the microfluidic upstream channel and each inlet reservoir. This port also functioned as a passive lock that hold the flow at rest via fluid-air surface tension, which facilitated manual movement of the device even when cell attachment was not achieved completely. To demonstrate the practicability of the system, we conducted experiments and diffusion simulations on the effect of endocrine disruptors on germ cells. To this end, a bisphenol-A (BPA) concentration gradient was generated in the main channel of the system at BPA concentrations ranging from 120.8 μM to 79.3 μM, and the proliferation of GC-1 cells in the BPA gradient environment was quantitatively evaluated. The features and concepts of the introduced design is to minimize unexpected and ignored error sources, which will be one of the issues to be considered in the development of microfluidic systems to explore extremely delicate cellular phenomena.

**Supplementary Information:**

The online version contains supplementary material available at 10.1186/s13036-023-00369-1.

## Introduction

A microfluidic system is an application of microelectromechanical systems (MEMS) technology whereby a fluidic-based experiment process is integrated at the microscale on a chip. This tool used for quantitative analysis and high-throughput assays has advantages such as low reagent consumption, low cost, and fast processing [1]. Especially, biochemical/mechanical stimuli are precisely controlled in the microfluidic chip to mimic the in vivo environment. Therefore, microfluidic-based cell culture devices, commonly referred to as organ-on-a-chip, are much better for observing the in vivo biological characteristics of cells than using conventional cell culture devices such as Petri dishes [1].

The extremely slow velocity (0.1–35 μm/s) in the microfluidic channel mimics the fluid flow in the interstitial space between blood vessels and controls the shear stress and molecular transport similar to the in vivo cell microenvironment [2]. The interstitial flow generated in the microfluidic devices can significantly influence the expression of the cell characteristics, which has been exploited in research on various human body phenomena such as cancer microenvironment [3, 4], neuronal cell differentiation [5, 6], angiogenesis [7–9], etc. The extremely slow laminar flow due to the microscale of the microfluidic system provides diffusion-predominant molecular mixing, which is a useful phenomenon for reproducing the in vivo biomolecular concentration. The molecular transport in the fluid is quantified by Péclet number (*Pe*), which is the ratio of convection and diffusion and a major design criterion for determining the flow condition of the microfluidic chip:

1 $$Pe= \frac{{v}_{s}L}{D}$$

where *v*_*s*_ is the average flow velocity, *L* is the characteristic length, and *D* is the diffusivity of the fluid. To mimic in vivo biomolecule transport using the microfluidic system, it is essential to satisfy the flow condition of *Pe* < 1 [10]. The diffusion-predominant molecular mixing maintains a stable concentration gradient of the biochemical in the microfluidic systems, which has been utilized in drug screening [1].

The Y-channel (or T-channel) is the usually used microfluidic gradient generator. The simple design of the Y-channel has the advantage of minimizing blocking and leakage during setup and experimentation [11]. Solutions of two different concentrations can enter separately through the two inlet ports and then form a gradient in the single channel. This can be used to provide a suitable cell culture medium [12] and the biochemicals involved in cell growth and differentiation [5, 13, 14], as well as maintaining the temperature of the medium [15]. Gradient chips have been used with various cell types, such as mesenchymal stem cells [12], neural progenitor cells [5], adipose-derived stem cells [13], lung epithelial cells [14], and drosophila embryos [15], among others. However, these previous achievements are still limited to functional validation in the laboratories.

**Fig. 1:**
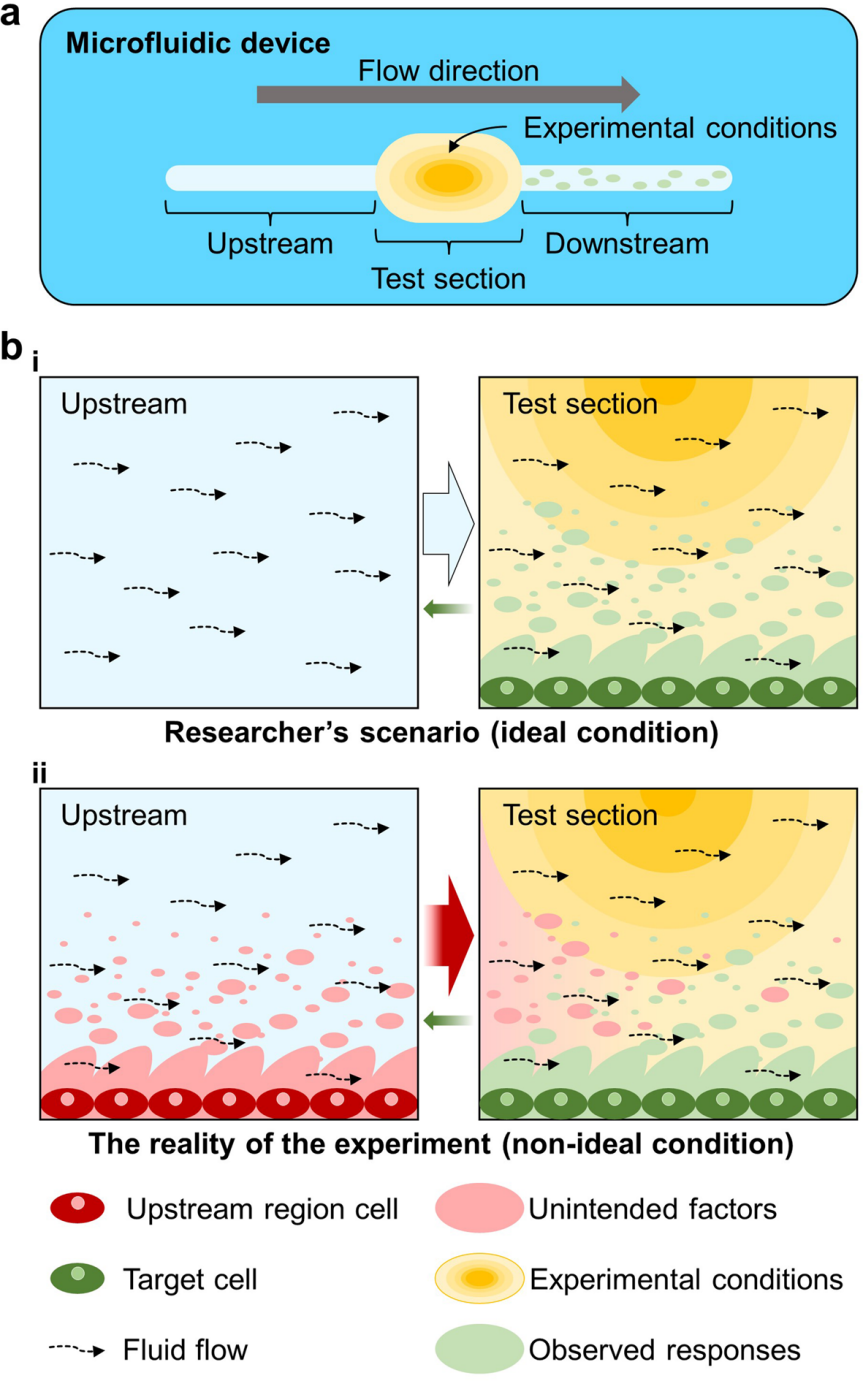
Schematic of the impact of the uncontrolled cells in upstream region on the main test section in microfluidic device. **a** Structure of a general microfluidic device. There is a microfluidic channel constituting the upstream and downstream of the test section for material exchange including the culture medium. **b** Influence of upstream cells on the experimental conditions in the test section. The ideal conditions researchers expect to design microfluidic devices and the non-ideal conditions in which cells cultured in the upstream region affect the test section

Currently, both developers and end-users agree that the technology of organ-on-a-chip, which is an integration of microfluidic systems, has not reached the level of industrialization, however, they evaluate the maturity of this technology differently [16]. In a survey on EU Horizon 2020 technology readiness levels (TRL) consisting of nine levels, end-users focused their opinions on 'TRL 4-technology validated in the laboratory', while most developer gathered opinions on 'TRL 7-system prototype demonstration in an operational environment'. Their opinion difference on maturity of organ-on-a-chip technology mean that developers need to take a closer look at the environment in which end-users operate their devices. In this respect, we paid attention to increase of the practicability of the system and reduction of human error. For various cell studies, the microfluidic device included a test section setting up the conditions of the experiments, and the test section was connected to microfluidic channels on the upstream and downstream sides (Fig. 1a). The users of these devices often limit their experimental conditions to the test section and omit the circumstances of the upstream and the downstream (Fig. 1b, i). However, in the reality of the experiment, the expected condition of the test section is influenced by its upstream (Fig. 1b, ii). The cells introduced in the form of a cellular suspension become attached to the bottom of the channel through which they pass, and the cells cultured in some these areas are not the experimental target. Moreover, these cells can secrete biomolecules that are delivered to the target cells through the flow in the microfluidic chip, which cause confusion in experimental data. Meanwhile, in the experimental site, researchers inevitably manually transfer the cell experimental tools based on microfluidics from a bench to a microscope or incubator. During this process, it is difficult to keep the device level and gravity-driven flow is generated when it is tilted. This unintended flow during the experiment destabilizes cell adhesion to the bottom of the device. Although it is anticipated that the engineers working in the laboratory were already aware of this situation, there are few reports on these problems and most studies concentrate on the main functions of microfluidic devices. However, since microfluidic technology has been applied to more precise cell research, it is necessary to overcome this minor problem that interferes with the accuracy of experimental results. To control the cell position and the flow inside the microfluidic chip, various techniques, such as the microvalve [17], encapsulation [18], sorting [19], trapping [20], isolation [21], etc., have been reported. However, the configuration of the microfluidic chip to which these technologies are applied increased the complexity of the microfluidic chip and difficulty of manufacturing the device. Moreover, the additional control facilities were required to operate this technology, which requires skill from the user of the device.

In this paper, we introduced a perspective on the design of a microfluidic devices for the purpose of applying physical phenomena by simple structural modifies that can be implemented with fabrication technology in common laboratories to solve the aforementioned practical issues. In detail, utilization of a narrow port structure installed between the microfluidic channel and each reservoir was emphasized to reduce unexpected interference of the cellular molecules delivered from the inlet reservoir and the handling error during the cell attachment. The delicate design was applied to the conventional gradient chip system operated by osmotic pump which has been previously proposed for cell-based research [5, 12]. A soft lithography-based method was applied to fabricate the improved gradient chip whereby the microfluidic channel and reservoirs are cast on a single piece of polydimethylsiloxane (PDMS). Theoretical calculations and numerical analysis were conducted to confirm that the port of the improved design is effective at reducing hydrodynamic instability that occurred during device handling and the effect of unnecessary cells cultured in the reservoirs.

Furthermore, we demonstrated the application of the gradient chip for endocrine disruptors (EDs) test on testis cells cultured in our system. EDs accumulate in the human body and interfere with normal homeostatic control, reproduction, and developmental processes [22]. Especially, it is known that one of the EDs that is frequently used in the manufacture of various plastics, bisphenol-A (BPA), is easily internalized in the body. In this experiment, GC-1 cells (an immortalized cell line derived from mouse testes) were cultured in our microfluidic system with a BPA concentration gradient created in the main channel, in which cell proliferation was observed. Only a few groups have previously considered the microfluidic-based platforms to investigate the physiology of testis [23–25], however, this has only focused on the study of spermatogenesis, and drug testing using microfluidics is currently insufficient. Therefore, this analysis using the gradient chip suggests that the application of microfluidic technology can lead to efficient experiments in testicular-related cell drug testing.

## Materials and methods

### Microfluidic chip fabrication

**Fig. 2:**
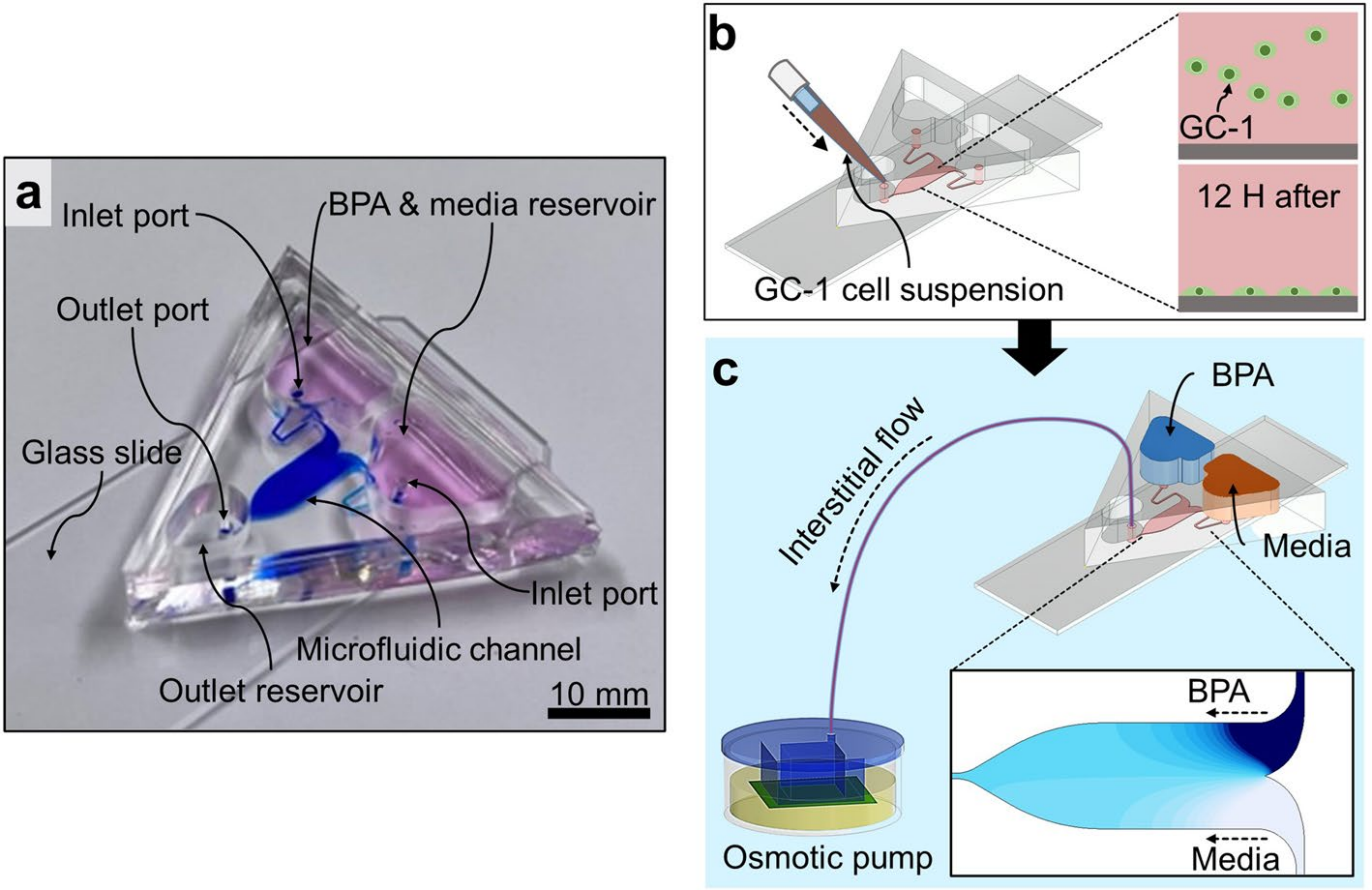
The structure and operation of the gradient chip. **a** An image of the gradient chip with the narrow port structure. Scale bar is 10 mm. **b** Injection of GC-1 cell suspension through the outlet port. In the static state, the floating cells go down and are attached to the channel bottom. **c** The generation of extremely slow flow using the osmotic pump. Each inlet reservoir is filled with the BPA solution and culture media, and then the gradient of BPA concentration is formed in the microfluidic channel

The microfluidic chip consists of a glass slide and a piece of PDMS (Sylgard® 184, Dow Inc., Midland, MI, US). The PDMS part provides the microfluidic channel (4 mm wide and 150 μm high), two inlet reservoirs, two inlet ports, an outlet port, and an outlet reservoir (Fig. 2a). In this system, BPA and the culture medium are separately contained in the inlet reservoir and then flow into the microfluidic channel. Two molds were used to cast the PDMS part of the microfluidic chip (supplementary figure S1). Using a computer numerical control machine (DAVID 3040, David Motion Technology, Incheon, Republic of Korea), the one for the reservoir made from acrylic was carved (supplementary figure S1). The PDMS solution was prepared in a 10:1 ratio of prepolymer and a curing agent and poured into the acrylic mold. After degassing in a vacuum chamber, the PDMS solution in the acrylic mold was cured for 2 h at 80℃, and the cured PDMS was separated from the acrylic mold. To use the cured PDMS as a mold, the PDMS surface was treated air plasma for 30 s, dipped in 70% ethanol solution for 10 min, and dried out. This serial treatment, called the double casting process, prevented the cured PDMS part and the added PDMS solution from combining during the PDMS curing [26]. The microfluidic channel of the PDMS part was cast using an SU-8 mold fabricated by photolithography (supplementary figure S2). Uncured PDMS solution was poured into the SU-8 mold, and after degassing in a vacuum chamber, cured for 2 h at 80℃. The reservoir mold was placed on the cured PDMS covering the SU-8 mold, and the structures in the reservoir mold were aligned with the inlets and outlets of the SU-8 mold. Uncured PDMS was carefully added between the two molds, and the PDMS curing process (2 h at 80℃) was conducted to merge the added PDMS and previously cured PDMS part. The reservoir mold and the SU-8 mold were separated from the PDMS structure with the microfluidic channel and reservoirs. Elastic deformation of the reservoir mold composed of PDMS facilitated the dismantling process. To connect the microfluidic channel with the reservoirs, the inlet and outlet ports were stamped with a 1.5 mm diameter biopsy punch. The PDMS part and the glass slide were treated with air plasma for 30 s and allowed to contract at 80℃ for 1 h for bonding to each other.

### Cell culture in the platform

GC-1 cells were grown in the culture media solution consisting of phenol red-free Dulbecco’s modified Eagle’s medium (LM 001–10, WELGENE, South Korea) supplemented with 1% fetal bovine serum supplemented, 1% penicillin and streptomycin, and 1 mM sodium pyruvate on a culture plate for a week. The gradient chip was sterilized by autoclaving at 121℃ and 15 psi for 15 min. To enhance cell attachment, the glass surface of the gradient chip was coated with 0.1% gelatin solution (gelatin from bovine skin, G9391, Sigma-Aldrich, St. Louis, MO, USA) for 30 min. Next, 20 µL of GC-1 cell suspension at a concentration of 1 × 10^6^ /mL cells was injected through the outlet port, and the cells in the chip were incubated under 5% CO_2_ at 37℃ for 12 h (Fig. 2b). The following day, an osmotic pump was connected to the outlet port to generate the interstitial level flow in the microfluidic channel, and the culture media solution and 200 μM BPA solution (239,658, Sigma-Aldrich, St. Louis, MO, USA) were loaded on the inlet reservoir, respectively (the strategy for filling the reservoir with the solution without air bubbles is explained in the supplementary video). The GC-1 cells were exposed to the BPA concentration gradient for 48 h (Fig. 2c). The experimental results were compared with those obtained from GC-1 cells cultured in a dish under the same conditions. Briefly, GC-1 cells were incubated under 5% CO_2_ at 37℃ for 12 h and then treated with BPA at concentrations of 0, 100, and 200 μM for 48 h. Subsequently, the treated cells were collected and counted by using the trypan blue exclusion assay.

### Operation of the gradient chip and the osmotic pump

The osmotic pump consists of a water cube, a cellophane membrane, and a polyethylene glycol (PEG) chamber (supplementary figure S3a). The water cube and the PEG reservoir contain deionized (DI) water and 0.01 M PEG solution (PEG 1000, DAEJUNG CHEMICALS & METALS Co., Republic of Korea), respectively, separated by the cellophane membrane, which allows only water molecules to pass through (supplementary figure S3b), thereby providing an extremely slow flow rate. For three trials, the transferred volumes of water were measured for more than 36 h (supplementary figure S3c). On average, the osmotic pumps transferred 18.55 μL of water for 36 h. The average mass flow measured at 24 h was 0.342 μL/h, which was applied as the flow condition in the flow simulation. This flow analysis result was compared with the simulation result under the condition of 3.42 μL/h to confirm the effect of the inlet port according to the flow rate. Since the flow condition of 0.342 μL/h in the gradient chip mimics the interstitial flow of *Pe* = 0.2375, the experiment applying this osmotic pump condition was performed.

### Numerical analysis

This was performed to demonstrate the utilization of the ports connected to the reservoirs and to predict the BPA concentration gradient in the chip. The ANSYS Fluent 19.2 (ANSYS, Inc., Canonsburg, PA, US) commercial computational fluid dynamic (CFD) tool, was used for this endeavor. The diffusion phenomenon was predicted using the species transport model. The conservation equation of the laminar species transport model and the diffusion flux based on Fick’s law are respectively as follows [27]:

2 $$\frac{\partial }{\partial t}\left(\rho {Y}_{i}\right)+\nabla \bullet \left(\rho \overrightarrow{v}{Y}_{i}\right)=\nabla \bullet {\overrightarrow{J}}_{i}$$

3 $${\overrightarrow{J}}_{i}=-\rho {D}_{i,m}\nabla {Y}_{i}$$

where *ρ* is the fluid density, *Y*_*i*_ is the local mass fractions of species *I*, $\overrightarrow{v}$ is the velocity component, ${\overrightarrow{J}}_{i}$ is the diffusion flux values for species *I*, and *D*_*i,m*_ is the mass diffusion coefficient for species *i*. The momentum of laminar flow can be expressed as

4 $$\frac{\partial \left(\rho \overrightarrow{v}\right)}{\partial t}+\nabla \bullet \left(\rho \overrightarrow{v}\overrightarrow{v}\right)=-\nabla \rho +\nabla \bullet \left(\tau \right)+\rho g+F$$

where *S*_*m*_ is the mass added from the source, *τ* is the stress component, *g* is the gravitational acceleration, and *F* is the force component.

**Fig. 3:**
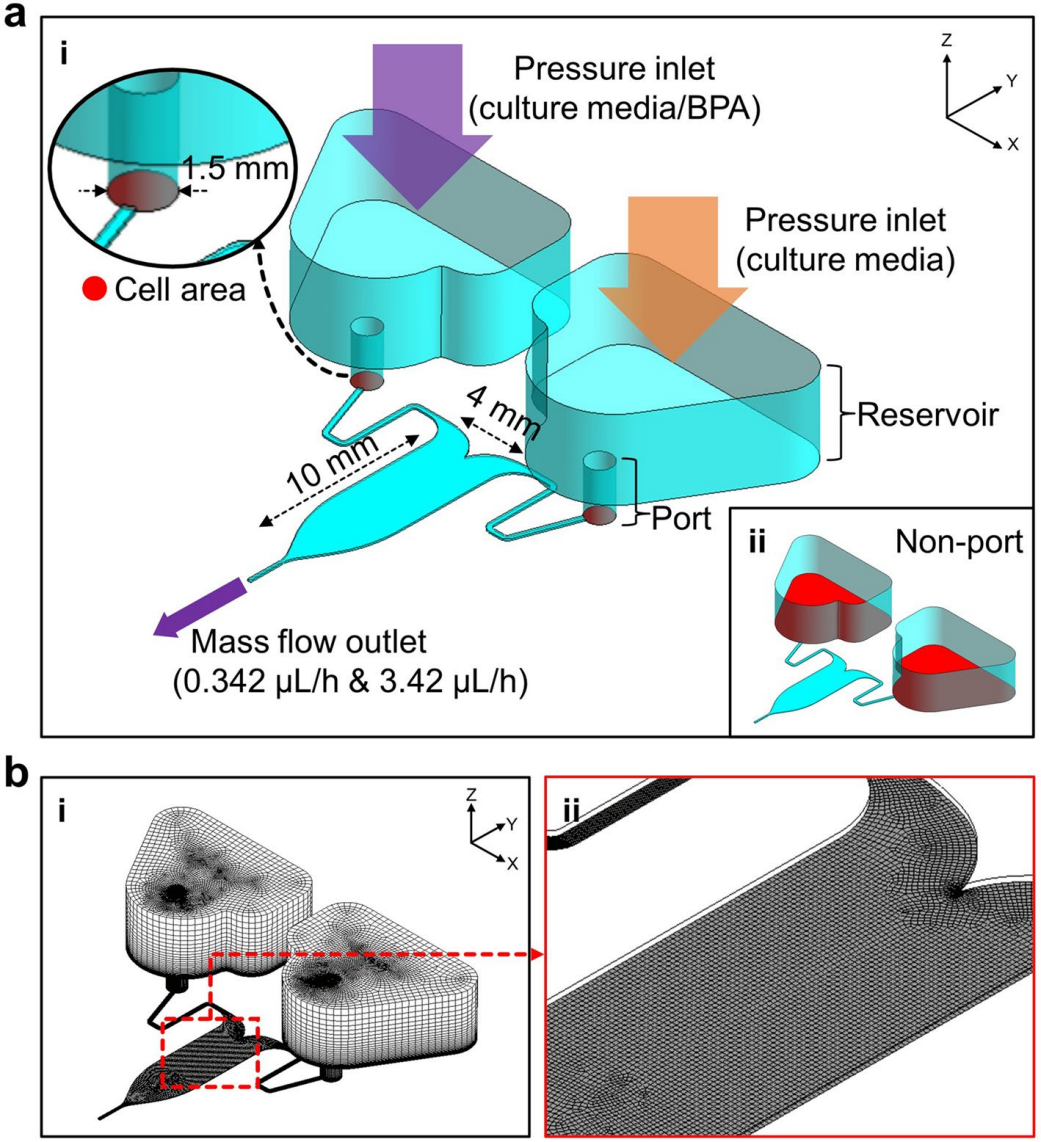
The computational model for the gradient chip. **a** The simulation was set up to validate the reservoir improvement and schematics of the gradient chips (i) with inlet ports and (ii) without inlet ports. **b** The mesh structure of the computational domain: (i) an overall view of the used hexahedral mesh and enlarged views of (ii) the area near the outlet and the converged area of the two inlet channels

First, it was assumed that biomolecules were continuously secreted from cells in the storage area and flowed into the main test section. The main test section of the gradient chip is 100 μm high, 10 mm long, and 4 mm wide, and the microfluidic channel was connected with two inlet ports 1.5 mm in diameter (Fig. 3a, i). At the bottom of ports, biomolecules with a mass fraction of 10% were allowed to diffuse at 1 × 10^–10^ m^2^·s^−1^. As the working fluid, water at 37℃ as an incompressible Newtonian fluid with a density of 993.3 kg·m^−3^ was used as the culture medium [28]. The analysis was performed at a mass flow rate of 0.342 μL/h (the average flow rate measured in the experiment) and 3.42 μL/h (supplementary figure S3c). A transient simulation was performed with a timestep of 3,600 s calculated from 100 iterations. The inlets were assumed to be under atmospheric pressure (1 atm). This boundary condition was also applied to another geometrical arrangement including the reservoirs without the inlet ports (Fig. 3a, ii). In addition, the concentration gradient of the BPA formed in the channel was predicted. Since it is expected that the formation of BPA concentration gradient is independent of the port and reservoir design, the reservoirs and inlet ports were ignored in the geometry of the BPA diffusion simulation (supplementary figure S4). Steady-state simulation was performed with the outlet having a mass flow rate of 0.342 μL/h and using the diffusivity value of trypan blue (2.21 × 10^–10^ m^2^·s^−1^) to validate the CFD model [29]. The analysis result was compared with the flow experiment using trypan blue. Subsequently, the BPA concentration gradient was calculated by applying the diffusivity value of BPA (5.05 × 10^–10^ m^2^·s^−1^) [30]. Each case was repeated 5,000 times, which resulted in residuals of less than 10^–6^. The number of mesh elements was 114,900 (Fig. 3b, i). A hexahedral mesh structure was applied to the entire CFD domain, including the narrow outlet area and the merging area of the two inlets (Fig. 3b, ii).

## Results and discussion

### The biochemical and hydrodynamic errors of the gradient chip

**Fig. 4:**
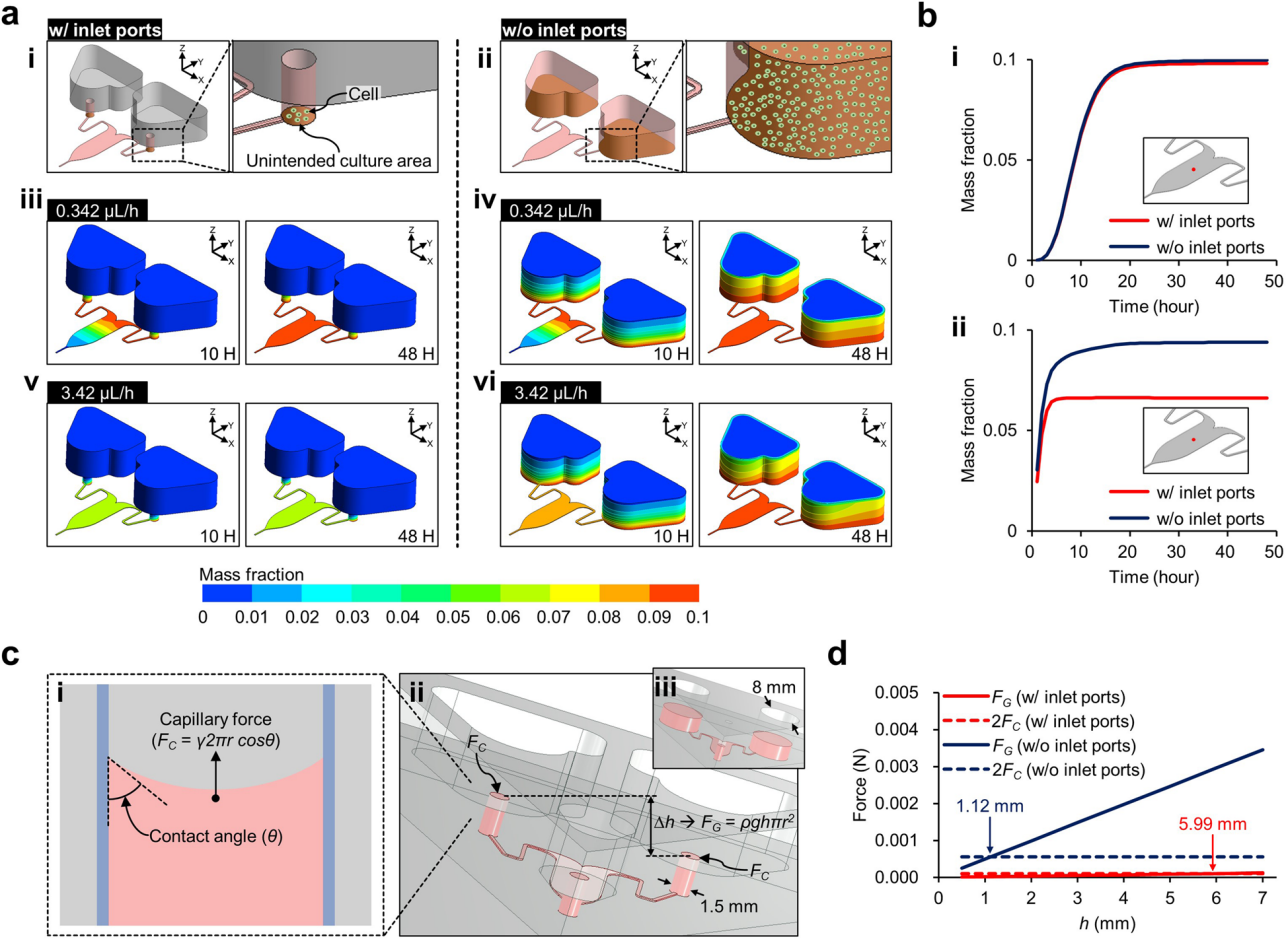
The functions of the inlet port. **a** The inflow of biomolecules cultured at the bottom of the gradient chip inlet. The unintended cell culture area of (i) the inlet port of the improved gradient chip and (ii) the reservoir without the port. Mass fraction contours of biomolecules under flow rate conditions of (iii and iv) 0.342 μL/h and (v and vi) 3.42 μL/h. **b** Mass fraction graphs measured at the center of the microfluidic channel (red colored point) under flow rates of (i) 0.342 μL/h and (ii) 3.42 μL/h. The red points in the inset images indicate where the mass fractions of the biomolecules were measured. **c** As a passive valve to prevent flow between the inlets during chip handling: schematics of (i) the capillary force on the inlet port fluid and (ii) the hydrostatic force generated by the height difference between the inlet ports of the inclined gradient chip. **d** A graph of the forces applied to the fluid in the gradient chip inlets

In several studies, the microfluidic channel is directly connected to the reservoir and shares the substrate at the bottom of the microfluidic chip [31, 32]. Thus, the cells become attached and cultured on the reservoir bottom. In the present experiment, the cell suspension was injected into the channel outlet and filled the entire channel, which evenly distributed the cells in the channel (Fig. 2b). Therefore, the cell suspension occupied the inlet port in the gradient chip (Fig. 4a, i). On the other hand, in the gradient chip without ports, the cells reached the reservoir and occupied the entire bottom of the reservoirs (Fig. 4a, ii). Since the condition of the reservoir without an inlet port is not a microfluidic environment, in this reservoir, cells were cultured in a 2D environment unintended for the utilization of this microfluidic system (The supplementary figure S5). The simulation was performed with flow rates of 0.342 μL/h and 3.42 μL/h. At a flow rate of 0.342 μL/h (Fig. 4a, iii and iv), the biomolecules were continuously diluted with the culture medium and introduced into the microfluidic channel during 10 h. The biomolecules had diffused through the outlet of the microfluidic channel, and at 48 h, the biomolecules were present over the entire area of the microfluidic channel in both gradient chips with and without ports. However, at a flow rate of 3.42 μL/h, since the cell secretion had been diluted with a larger amount of the culture media than at the lower flow rate, the biomolecules flowing into the channel were reduced (Fig. 4a, v and vi). Especially, in the gradient chip with the port, the biomolecules were diluted with the culture media, and the concentration was maintained for 48 h (Fig. 4a, v). In the gradient chip without ports, although the biomolecules had been diluted, their mass fraction gradually increased, and the reservoirs became considerably saturated at 48 h (Fig. 4a, vi). With a flow rate of 0.342 μL/h, the mass fraction of the biomolecules gradually increased and became saturated after 24 h (Fig. 4b, i). The saturated mass fractions in the gradient chip with and without ports were predicted to be 9. 81 × 10^–2^ and 9.97 × 10^–2^, respectively. These data show that the cells in the port affect the cells in the channel by 1.55% less than the cells in the reservoir without port. Although it was difficult to confirm this trend on the mass fraction contour (Fig. 4a, iii and iv), the quantitative data confirmed the positive effect of the inlet port. This reduction in unnecessary biomolecules became apparent as the flow rate was increased (Fig. 4b, ii). At a flow rate of 3.42 μL/h, the secretions from the cells cultured in the port became saturated with a mass fraction of 6.61 × 10^–2^ in the channel whereas in the gradient chip without ports, the saturated mass fraction increased by 29.5% (9.38 × 10^–2^). This CFD analysis indicates that more careful evaluation of the reality of the experimental procedure into the microfluidic chip design can improve the accuracy of the experiment. In conclusion, it was predicted that the benefit of the port would be more effective at higher flow rates. However, since the higher the flow rate inside the gradient chip, the sharper the concentration gradient is formed, researchers need to consider all of these issues when determining the flow rate of the channel [12].

This narrow port design compensated for another error factor in the microfluidic chip. We demonstrated the applicability of the gradient chip with its reservoirs open to the atmosphere to reduce hydrodynamic instability and interference from redundant cells cultured in the reservoirs. The capillary force in the port connecting the reservoir and the microfluidic channel functions as a passive valve to prevent gravity-driven flow (Fig. 4c). Two forces act on the fluid in the ports. The first is capillary force (*F*_*C*_) that holds the fluid in the port (Fig. 4c, i), which is defined as

5 $${F}_{C}=\gamma 2\pi rcos\theta$$

where *γ* is the surface tension (0.07 N/m) [33], *θ* is the contact angle (81°) [34], and *r* is the port radius. Using this equation, the *F*_*C*_ value of the fluid in the ports and reservoirs without ports were calculated as 0.103 and 0.550 mN, respectively. On the other hand, the gravity force (*F*_*G*_) applied to the fluid in the inclined chip is obtained as

6 $${F}_{G}=\rho g\Delta h\pi {r}^{2}$$

where *ρ* is the fluid density, *g* is the gravitational acceleration (9.81 m/s^2^), and *∆h* is the height difference between the top surface of the fluid in the ports (Fig. 4c, ii). The reservoirs contain a greater weight of fluid in the gradient chip without ports than in the one with ports (Fig. 4c, ii and iii). Depending on the *∆h* value, the *F*_*G*_ value of the fluid in the reservoirs of the chip without ports increases significantly compared to the force in the ports (Fig. 4d). In the ports, the fluid remains static when the capillary force is greater than the gravity, and derived from Eqs. (5) and (6), the height difference at which the *F*_*C*_ value and gravity are at equilibrium is calculated as

7 $$\Delta {h}_{E}=\frac{2\gamma cos\theta }{\rho gr}$$

In the gradient chip without ports (8 mm-diameter reservoirs), the *∆h*_*E*_ at which the *F*_*G*_ and the *F*_*C*_ values are balanced is 1.12 mm. However, in the inclined device with ports, the effect of capillary force is enhanced, and so *∆h*_*E*_ is increased to 5.99 mm. We observed the passive valve function by surface tension in a narrow port through experiments (supplementary figure S6). Consequently, the flow instability during chip handling is significantly inhibited by using the port as a passive valve.

We verified the effect of the port on cell distribution and flow stability, which suggests that, not only the cell manipulation technology, but also the delicate design of unnoticed geometric factors constituting the microfluidic chip should be considered for the precise realization of the experimental conditions of the microfluidic chip.

### The diffusion simulation of BPA concentration

**Fig. 5:**
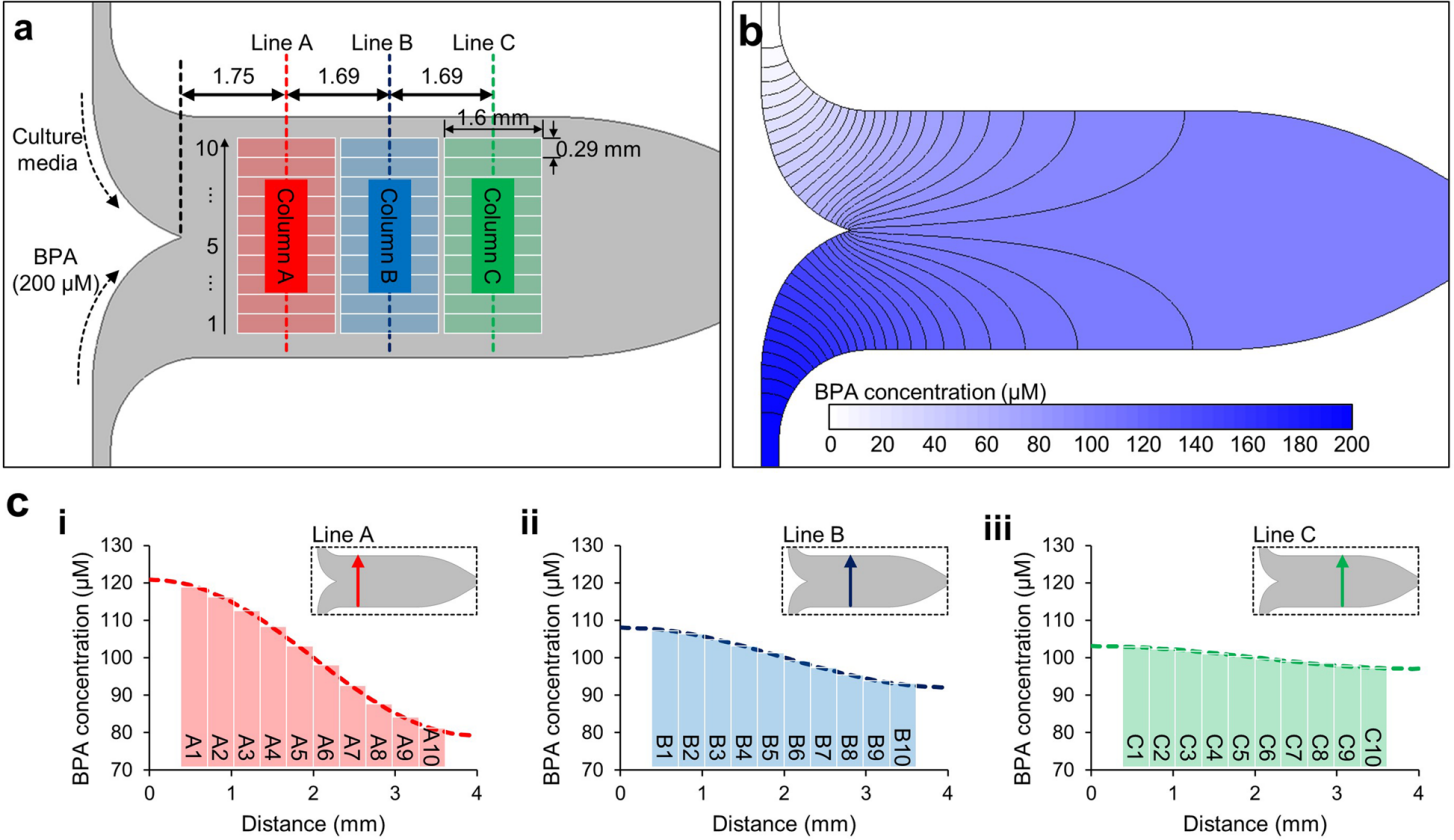
The diffusion simulation results. **a** Schematic of the gradient chip. The normal culture medium and the culture medium containing 200 uM BPA flow into each inlet of the gradient chip. The GC-1 cells were observed in the square Sects. (1.6 × 0.29 mm) with three columns (aligned on lines A, B, and C) and 10 rows. **b** The mass fraction contour of the BPA concentration in the gradient chip. **c** The graphs of BPA concentration (i) upstream, (ii) midstream, and (iii) downstream (lines A, B, and C in the insets, respectively)

We performed the diffusion simulation to predict the BPA concentration in the gradient chip. To verify our CFD model, the experiment was performed using trypan blue, which is easy to analyze with the naked eye. In the CFD model, the diffusion coefficient of trypan blue was applied, and the mass fraction contour confirmed in the CFD model predicted the color intensity of the trypan blue gradient similarly (supplementary figure S7). The gradient profile of the CFD model was consistent overall with the normalized intensity measured in the image (supplementary figure S7). Therefore, we used this CFD model to calculate the BPA concentration in the gradient chip (Fig. 5). The culture medium and 200 μM BPA solution were introduced through separate inlets (Fig. 5a). The profile of the BPA gradient was measured upstream (line A), midstream (line B), and downstream (line C) of the channel. The concentration contour shows that the BPA concentration gradient was formed in the cell culture area, and diffusion-mixing occurred gradually as the BPA flowed downstream (Fig. 5b). The diffusion-mixing reduced the concentration gradient in the flow; the BPA concentration ranges were calculated as 120.8–79.3 μM upstream, 108.0–92.1 μM downstream, and 103.1–97.1 μM upstream and was predicted as 100 μM at the center of each area (Fig. 5c, i–iii).

Since only an infinitesimal amount of BPA was required in the experiment, it is difficult to prepare several BPA solutions with slight differences in concentration. However, the concentration gradient created by this chip easily provides the experimental conditions required to test slight differences in BPA concentration simultaneously. In addition, it is possible to control the concentration range in the microfluidic channel by adjusting the concentration of the BPA solution loaded on the inlet reservoirs.

### Testing the effect of BPA concentration on GC-1 cells

**Fig. 6:**
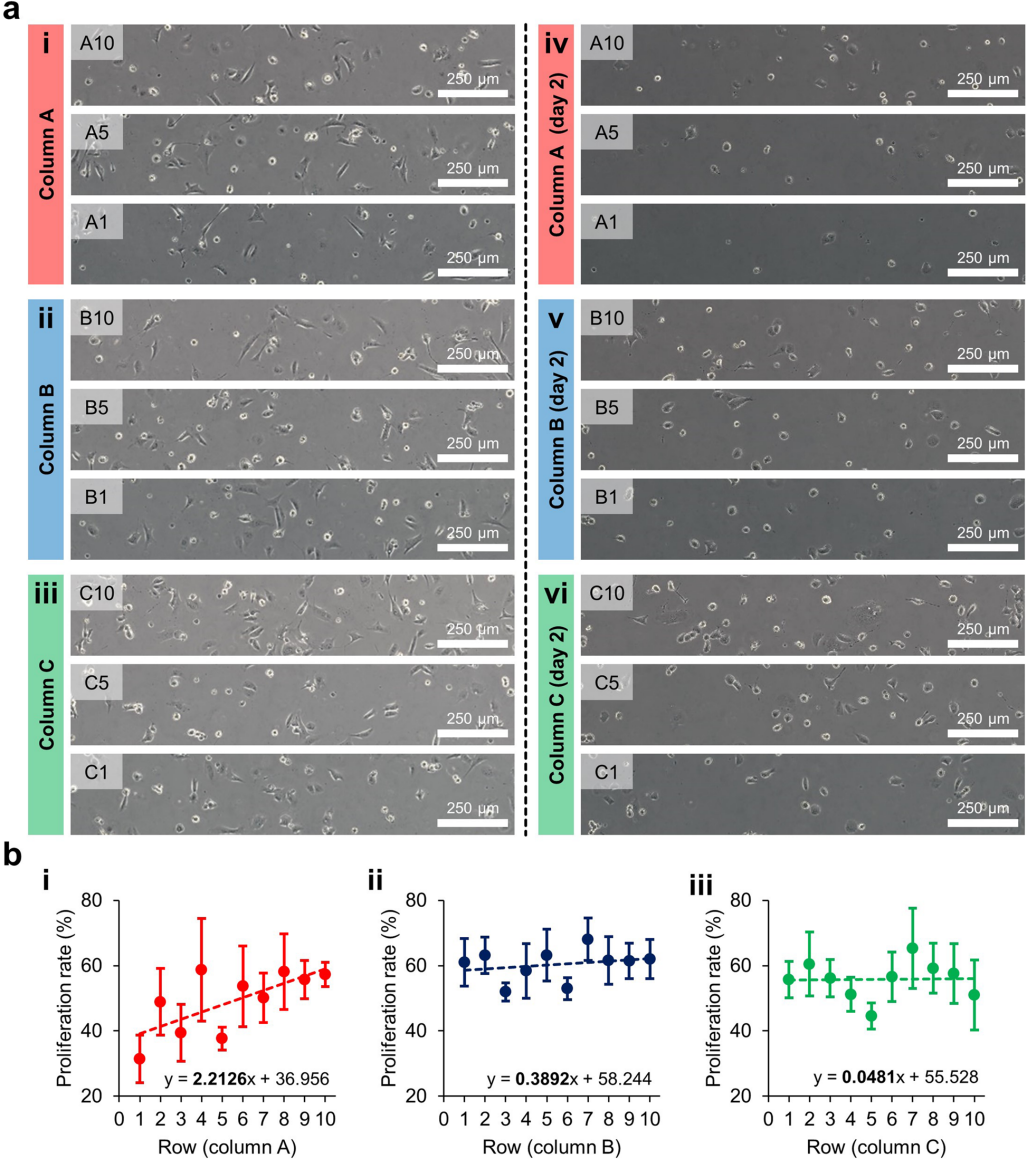
The effect of BPA on GC-1 cells in the gradient chip. **a** Bright field images of GC-1 cell reduction in the upstream region (sections A1, A5, and A10), the midstream region (section B1, B5, and B10), and the downstream region (sections C1, C5, and C10). **b** Graphs of the GC-1 cell proliferation rates observed in (i) the upstream region (column A), (ii) the midstream region (column B), and the downstream region (column C). Each equation in the graph indicates the linear trend (the dashed lines)

In the main test section where the GC-1 cells attached uniformly for 12 h (Fig. 6a, i–iii), 200 μM BPA was introduced in the channel through one of the inlets, then the laminar flow in the channel generated the BPA gradient. After exposure to the BPA gradient for 2 days, the GC-1 cells were damaged by the BPA (Fig. 6a, iv–vi); the cells had shrunk, and their pseudopodia had almost disappeared. In addition, it can be observed that the number of cells decreased depending on the BPA concentration in each section. Compared to columns B and C, the morphological alterations and reduction in the number of cells were prominent in column A (Fig. 6a, iv). From the results of the CFD analysis, the concentration difference in BPA between Sections A1 and A10 was 41.5 μM, and so the BPA gradient had obviously formed in column A (Fig. 5c, i). On the other hand, the BPA molecules were mixed as the flow proceeded downstream, and so columns B and C showed reductions of the concentration difference between rows 1 and 10 of 15.9 and 6.0 μM, respectively (Fig. 5c, ii and iii). The number of cells in each section was counted and the cell proliferation rate was then calculated (*n* = 4). The GC-1 cells were generally reduced by the BPA. Since the BPA gradient was formed steeper in the upstream (Fig. 5c), the decrease in cells according to the BPA concentration was also evident in column A, corresponding to the upstream region (Fig. 6b). In the proliferation rate graph, the slopes of the trend lines was calculated as 2.2126, 0.3892, 0.0481 for columns A, B, and C, respectively. The experiment using the gradient chip facilitated cell observation in a designated local area. Therefore, the morphological adjustment of GC-1 cells could be directly traced, and the data provided by the CFD technique supported the analysis of the cell proliferation depending on the BPA concentration. Although many biological studies have been conducted on the microcellular environment of the testis from which GC-1 is derived, more research on the mechanical phenomena in this tissue needs to be performed. This study is expected to contribute to the diversification of testicular cell-related experimental techniques through the application of a microfluidic chip. Determination of optimal conditions to simulate the testis microenvironment is considered a challenge to be achieved with this experimental methodology in the future.

**Fig. 7:**
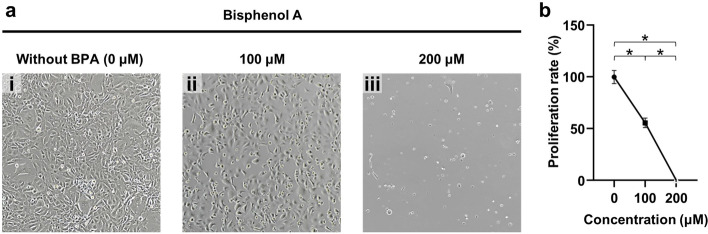
The effect of BPA on GC-1 cells in 2D culture conditions. **a** Images showing the effects of BPA according to concentration. **b** A graph of the proliferation rate of CG-1 cells exposed to BPA. The proliferation rate is the ratio of the harvested GC-1 cells after culturing to the number of intact GC-1 cells after treatment with BPA for 48 h (*P* < 0.05)

For comparison, the effect of BPA on the GC-1 cells cultured in a static 2D environment was tested. CG-1 cells were cultured with 0, 100, and 200 μM BPA. In the brightfield images in Fig. 7a, it can be observed that the GC-1 cells were damaged by the BPA. These results show that both 100 and 200 μM BPA were toxic to the GC-1 cells, and especially, most of the cells had become detached from the bottom when dosed with 200 μM BPA. The proliferation rates were 55.7% in 100 μM BPA and 0.27% in 200 μM BPA, the latter being extremely low (Fig. 7b). In the gradient chip, the middle of the channel (between rows 5 and 6 in columns A, B, and C in Fig. 5a) is the area where a concentration of 100 μM BPA formed (Fig. 5c), and on average, the cell proliferation rate was estimated in the middle of the channel of the trend line at 55.1%, which is 1.08% different from the value obtained from the static culture test. It was confirmed that the toxicity of BPA to statically cultured GC-1 cells was maintained even in cells in a microfluidic environment.

### Potential applications of the EDs study and challenges

Continuing exposure to EDs deleteriously affects thyroid function, corticosteroid production, and nervous system function in the human body [35–37]. EDs are present in synthetic chemicals such as solvents, lubricants, plastics, and pharmaceuticals. Especially, BPA, polybrominated biphenyls, polychlorinated biphenyls, dichlorodiphenylltrichloroethane, dioxin, polychlorinated biphenyls, phthalate esters, Endosulfan, Zeranol, and Atrazine are known to cause conditions such as cancer and infertility [36, 38]. Our experimental methodology accurately predicted and measured the effect of concentration of BPA on GC-1 cell efficiently in a single microfluidic chip. This is the first trial using our microfluidic chip system to explore the harmfulness of BPA on GC-1 cells. Thus, determining the harmful concentration not only of BPA but also of other EDs can be determined using our microfluidic chip system. Therefore, we expect that our approach can be utilized in the study of reproductive diseases caused by EDs.

Although functional improvement of the microfluidic chip was provided by our design, other improvements could enhance its practicability. For example, although using a reservoir port stabilized the flow of the culture medium during cell loading, flow instability was still present during the entire experimental process. Furthermore, in the channel, the interstitial flow depends on osmosis, which makes it difficult to consistently maintain the flow conditions during the entire experimental period.

## Conclusions

Microfluidic platforms including the gradient generator are playing an increasingly pivotal role in cellular studies, and their advantages and potential have been widely reported. However, as the diversity of utilizations and the number of users increase, erroneous phenomena that the engineers of the system did not foresee are becoming more noticeable. Even more worrisome is the potential for such errors to go unnoticed during the experiment, thereby producing erroneous data. Therefore, it is the right time to improve microfluidic systems by considering and applying the physical and chemical phenomena that predominate at the microscale. In this study, we showed how appropriate engineering can reduce the flow instability during the cell attachment and unwanted biomolecules in the inlet reservoir flowing into the test section. The reservoir structure open to the atmosphere was the main improvement design feature. The port added to the inlet reservoir holds the interface between the air and fluid, maintaining the stationary state of the culture medium by capillary force during the handling that causes tilting the gradient chip. It was confirmed that the flow stability was maintained at an inlet height difference (*∆h*) of less than 5.99 mm when the chip was inclined. Furthermore, the area reduction of the reservoir bottom (Fig. 5c) minimized redundant biomolecules flowing into the test section of the gradient chip. In the improved gradient chip, biomolecules were reduced by 1.55% and 29.5% at flow rates of 0.342 and 3.42 μL/h, respectively. To demonstrate the practicality of this system, we applied the device to investigate the effect of BPA on the GC-1 cells. The BPA concentration gradient was formed in the main test section where the GC-1 cells were cultured. The BPA concentration in the gradient chip was numerically predicted using CFD simulation to quantitatively evaluate cell proliferation dependent on the BPA concentration. By comparing the cell proliferation data of the 100 μM BPA area in the gradient chip with that of statically cultured GC-1 cells exposed to the same concentration, the reliability of our gradient chip system was verified. Engineering considerations to minimize the possible erroneous causes and their impact on microfluidic system design were introduced. Such efforts in engineering improvement are regarded as essential for sophisticated and delicate experiments with germ cells demonstrated in this paper. The scope of microfluidic systems that has grown well so far will be further expanded for in-depth research into cell biology and tissue engineering via more engineering improvements in the future.

### Supplementary Information

**Additional file 1.** Video.

**Additional file 2: Figure S1.** Reservoir mold fabrication using CNC milling and double casting. (a) Fabrication process of the reservoir mold. The images of (b) acrylic mold and (c) PDMS reservoir mold. **Figure S2**. Soft lithography and plasma-bonding of the microfluidic chip using the SU-8 mold and PDMS reservoir mold. **Figure S3**. Interstitial level flow generation using the osmotic pump. (a) The configuration of the osmotic pump, (b) water molecule transfer of the osmosis, and (c) flow measurements of the osmotic pump. **Figure S4**. A schematic of the diffusion gradient generation simulation. (a) The geometry and boundary condition of the simulation model. (b) The grid density of port design geometry was equally applied. **Figure S5**. Minimization of reservoir cells by inlet port structure. The images of the cells in the channel with (a) 1.5 mm diameter inlet ports with improved reservoir geometry and (b) the channel with 8 mm diameter reservoirs without inlet port. Photographs were taken 10 h after cell seeding. On the bottom of the inlet port, significantly fewer cells grew compared to the conventional channel including the 8 mm diameter reservoir without port. The area of the 8 mm diameter reservoir bottom was approximately 28 times larger than the area of the 1.5 mm diameter inlet port. Thus, with a smaller inlet port area, our system was able to minimize cells in the reservoirs that could have unwanted effects downstream. Scale bars are 1 mm. **Figure S6**. Flow stability by inlet port structure. (a) Experimental setup. A microscope with a channel was tilted by an acrylic slope of 10° and 20°. (b) Observation region. The flow occurred to the tilt direction. (c) Movement of cells by flow generated in the microfluidic channel. **Figure S7**. Validation of the CFD model. (a) A photographic image of a Trypan blue concentration gradient, (b) the contour of the Trypan blue mass fraction predicted by the CFD model, and (c) the comparison of the experimental data and CFD result on the red line in the inset. The color in the gradient chip along the red line in the inset was measured and normalized to compare with the mass fraction data of the CFD result.

## Data Availability

All data generated or analyzed during this study are included in this published article.
