# Supplementary material for: Fluid dynamic design for mitigating undesired cell effects and its application to testis cell response testing to endocrine disruptors
Source: J Biol Eng. 2023 Aug 7;17:51. doi: 10.1186/s13036-023-00369-1 (PMC10408176; doi:10.1186/s13036-023-00369-1)
Supplement: Supplementary file 2 — Additional file 2: Figure S1. Reservoir mold fabrication using CNC milling and double casting. (a) Fabrication process of the reservoir mold. The images of (b) acrylic mold and (c) PDMS reservoir mold. Figure S2. Soft lithography and plasma-bonding of the microfluidic chip using the SU-8 mold and PDMS reservoir mold. Figure S3. Interstitial level flow generation using the osmotic pump. (a) The configuration of the osmotic pump, (b) water molecule transfer of the osmosis, and (c) flow measurements of the osmotic pump. Figure S4. A schematic of the diffusion gradient generation simulation. (a) The geometry and boundary condition of the simulation model. (b) The grid density of port design geometry was equally applied. Figure S5. Minimization of reservoir cells by inlet port structure. The images of the cells in the channel with (a) 1.5 mm diameter inlet ports with improved reservoir geometry and (b) the channel with 8 mm diameter reservoirs without inlet port. Photographs were taken 10 h after cell seeding. On the bottom of the inlet port, significantly fewer cells grew compared to the conventional channel including the 8 mm diameter reservoir without port. The area of the 8 mm diameter reservoir bottom was approximately 28 times larger than the area of the 1.5 mm diameter inlet port. Thus, with a smaller inlet port area, our system was able to minimize cells in the reservoirs that could have unwanted effects downstream. Scale bars are 1 mm. Figure S6. Flow stability by inlet port structure. (a) Experimental setup. A microscope with a channel was tilted by an acrylic slope of 10° and 20°. (b) Observation region. The flow occurred to the tilt direction. (c) Movement of cells by flow generated in the microfluidic channel. Figure S7. Validation of the CFD model. (a) A photographic image of a Trypan blue concentration gradient, (b) the contour of the Trypan blue mass fraction predicted by the CFD model, and (c) the comparison of the experimental data and CFD result on the [file 13036_2023_369_MOESM2_ESM.pdf]

Supplementary Information

Fluid dynamic design for mitigating undesired cell effects and its application to testis cell response testing to endocrine disruptors

Seungjin Lee,†[a] Jinseop Ahn,†‡[b] Seok-Man Kim,[b] Daehan Kim,[a] Jiun Yeom,[a] Jeongmok Kim,[a] Joong Yull Park*[ac] and Buom-Yong Ryu*[b]

[a.] School of Mechanical Engineering, College of Engineering, Chung-Ang University, Seoul 06974, Republic of Korea
[b.] Department of Animal Science and Technology & BET Research Institute, Chung-Ang University, Anseong, 17546, Republic of Korea
[c.] Department of Intelligent Energy and Industry, Graduate School, Chung-Ang University, Seoul 06974, Republic of Korea
† Authors contributed equally.
‡ Current address: Columbia Center for Translational Immunology, Department of Medicine, Columbia University Irving Medical Center, New York, NY 10032, USA
*Corresponding authors contributed equally.

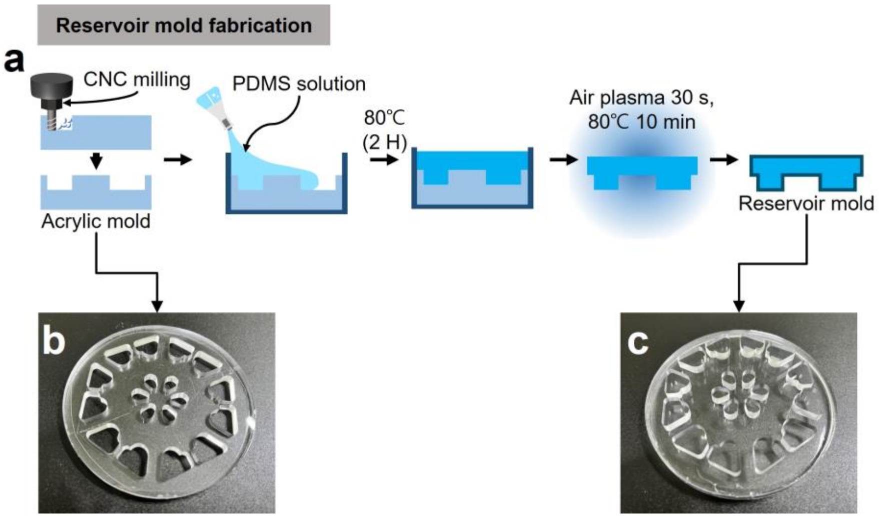

Figure S1. Reservoir mold fabrication using CNC milling and double casting. (a) Fabrication process of the reservoir mold. The images of (b) acrylic mold and (c) PDMS reservoir mold.

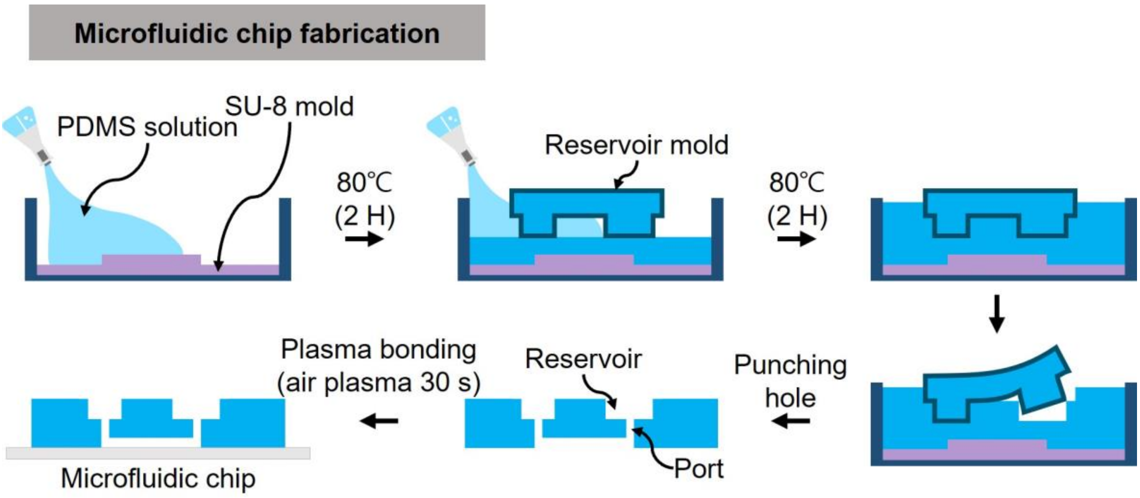

Figure S2. Soft lithography and plasma-bonding of the microfluidic chip using the SU-8 mold and PDMS reservoir mold.

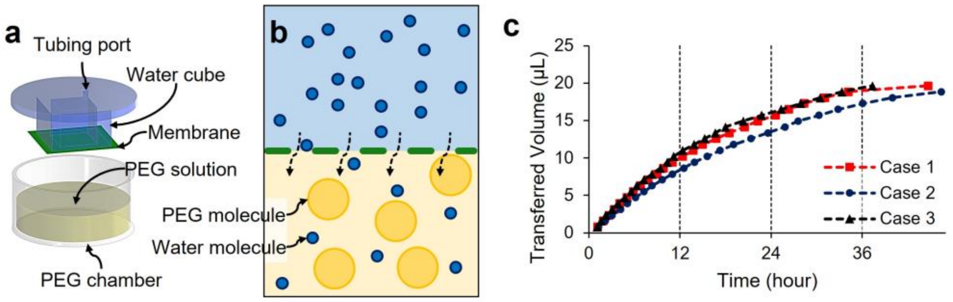

Figure S3. Interstitial level flow generation using the osmotic pump. (a) The configuration of the osmotic pump, (b) water molecule transfer of the osmosis, and (c) flow measurements of the osmotic pump.

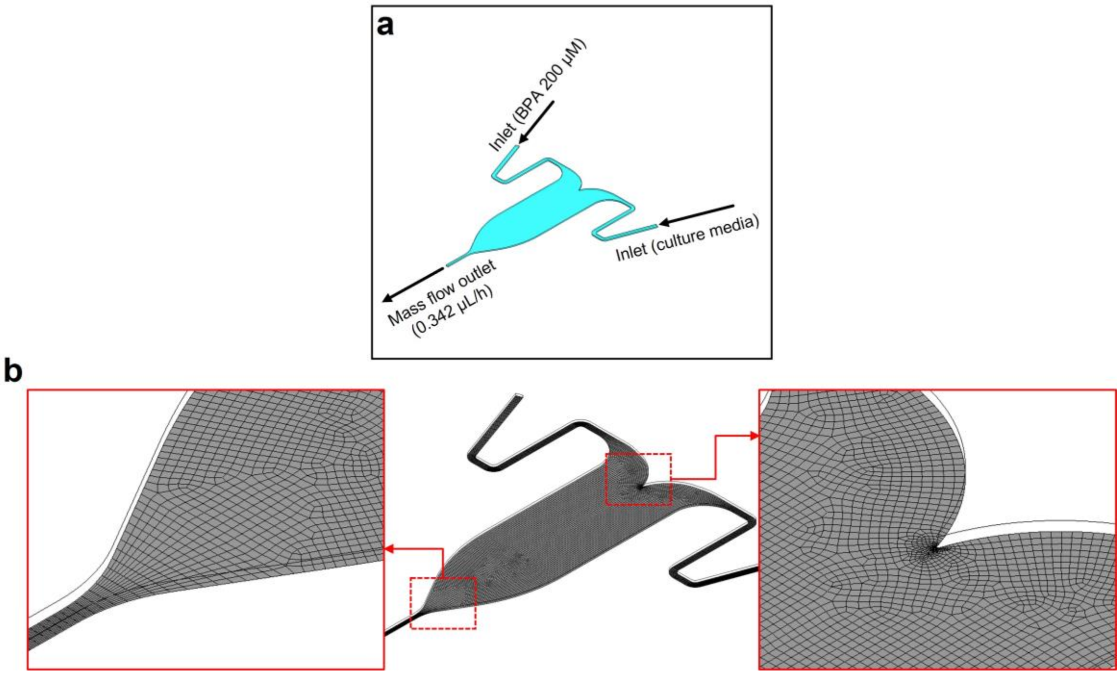

Figure S4. A schematic of the diffusion gradient generation simulation. (a) The geometry and boundary condition of the simulation model. (b) The grid density of port design geometry was equally applied.

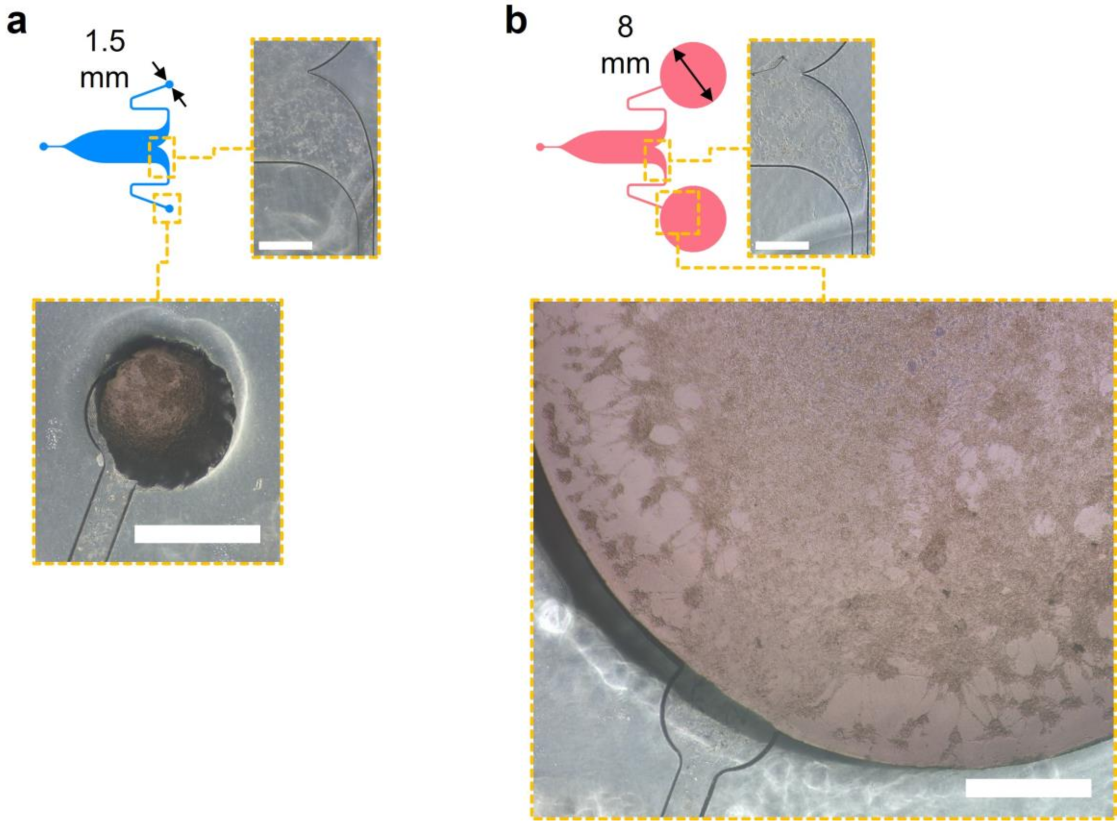

Figure S5 Minimization of reservoir cells by inlet port structure. The images of the cells in the channel with (a) 1.5 mm diameter inlet ports with improved reservoir geometry and (b) the channel with 8 mm diameter reservoirs without inlet port. Photographs were taken 10 h after cell seeding. On the bottom of the inlet port, significantly fewer cells grew compared to the conventional channel including the 8 mm diameter reservoir without port. The area of the 8 mm diameter reservoir bottom was approximately 28 times larger than the area of the 1.5 mm diameter inlet port. Thus, with a smaller inlet port area, our system was able to minimize cells in the reservoirs that could have unwanted effects downstream. Scale bars are 1 mm.

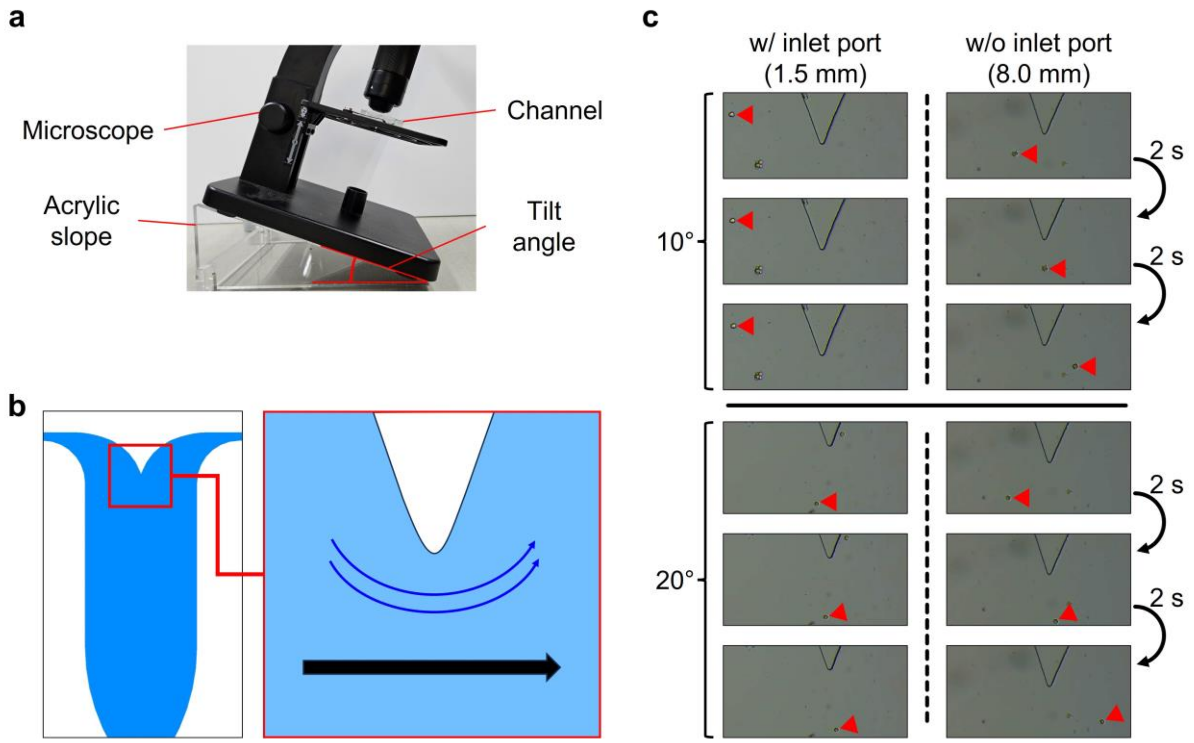

Figure S6 Flow stability by inlet port structure. (a) Experimental setup. A microscope with a channel was tilted by an acrylic slope of 10° and 20°. (b) Observation region. The flow occurred to the tilt direction. (c) Movement of cells by flow generated in the microfluidic channel.

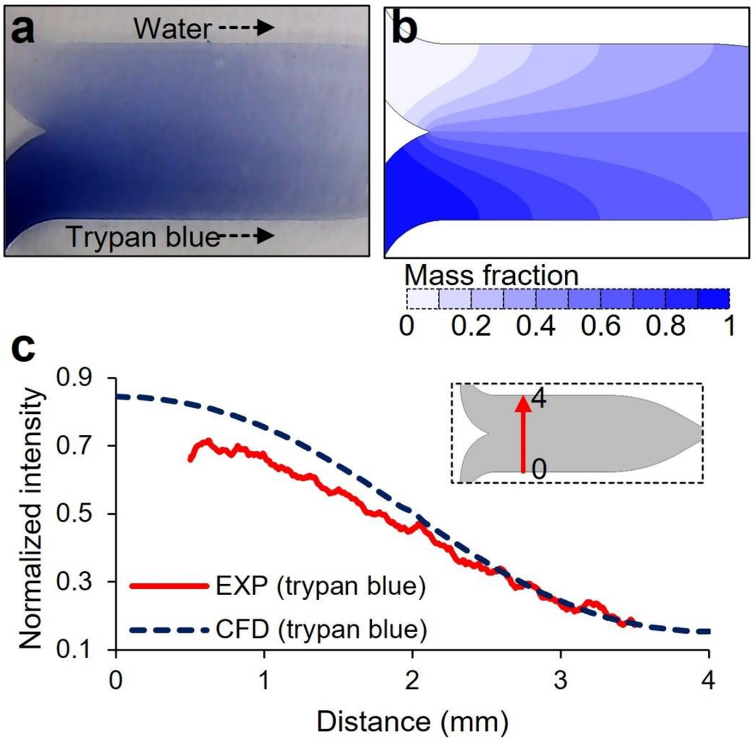

Figure S7. Validation of the CFD model. (a) A photographic image of a Trypan blue concentration gradient, (b) the contour of the Trypan blue mass fraction predicted by the CFD model, and (c) the comparison of the experimental data and CFD result on the red line in the inset. The color in the gradient chip along the red line in the inset was measured and normalized to compare with the mass fraction data of the CFD result.
